# Supplementary material for: The microbiome biomarkers of pregnant women’s vaginal area predict preterm prelabor rupture in Western China
Source: Front Cell Infect Microbiol. 2024 Oct 31;14:1471027. doi: 10.3389/fcimb.2024.1471027 (PMC11560878; doi:10.3389/fcimb.2024.1471027)
Supplement: Supplementary file 1 [file DataSheet1.zip › compare_1/Community/Pieplot/genus/sample/P18.genus.pieplot.pdf]

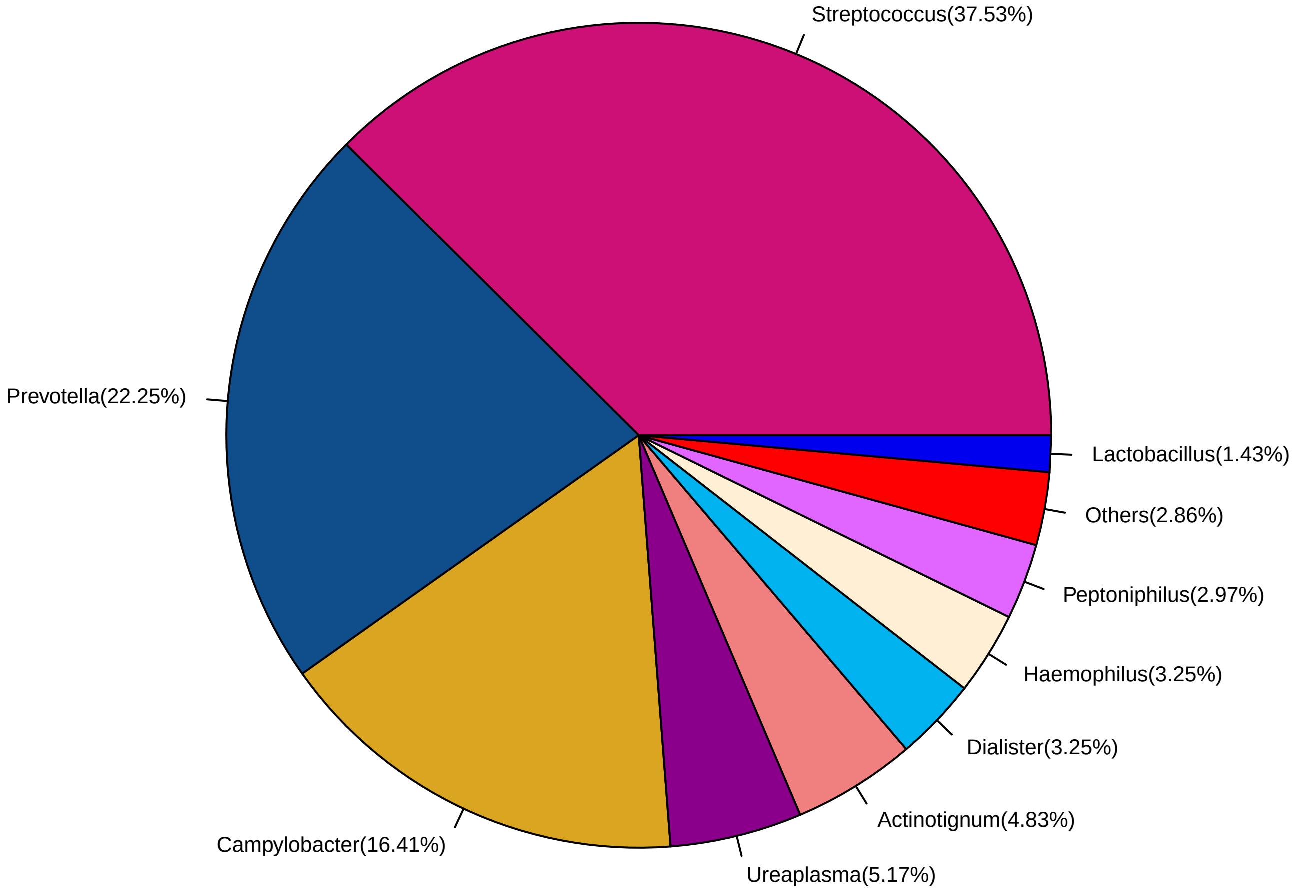
Streptococcus(37.53%)
Prevotella(22.25%)
Lactobacillus(1.43%)
Others(2.86%)
Peptoniphilus(2.97%)
Haemophilus(3.25%)
Dialister(3.25%)
Actinotignum(4.83%)
Ureaplasma(5.17%)
Campylobacter(16.41%)
